# Brown Tumour in a Patient with Secondary Hyperparathyroidism Resistant to Medical Therapy: Case Report on Successful Treatment after Subtotal Parathyroidectomy

**DOI:** 10.1155/2009/827652

**Published:** 2009-04-16

**Authors:** Nicola Di Daniele, Stefano Condò, Michele Ferrannini, Marta Bertoli, Valentina Rovella, Laura Di Renzo, Antonino De Lorenzo

**Affiliations:** ^1^Department of Internal Medicine, Tor Vergata University, Viale Oxford 81, 00133 Rome, Italy; ^2^Nephrology and Dialysis Unit, Tor Vergata University, Viale Oxford 81, 00133 Rome, Italy; ^3^Genetic Unit, Department of Biopathology and Imaging Diagnostic, Tor Vergata University, Viale Oxford 81, 00133 Rome, Italy; ^4^Department of Neuroscience, Division of Human Nutrition, Tor Vergata University, Viale Oxford 81, 00133 Rome, Italy

## Abstract

Brown tumour represents a serious complication of hyperparathyroidism. Differential diagnosis, based on histological examination, is only presumptive and clinical, radiological and laboratory data are necessary for definitive diagnosis. Here we describe a case of a brown tumour localised in the maxilla due to secondary hyperparathyroidism in a young women with chronic renal failure. Hemodialysis and pharmacological treatment were unsuccessful in controlling secondary hyperparathyroidism making it necessary to proceed with a subtotal parathyroidectomy. The proper timing of the parathyroidectomy and its favourable effect on regression of the brown tumor made it possible to avoid a potentially disfiguring surgical removal of the brown tumor.

## 1. Introduction

Secondary hyperparathyroidism is a frequent complication of chronic renal failure as a consequence of renal osteodystrophy. Brown tumours, as unifocal or multifocal bone lesions, represent a serious complication of advanced hyperparathyroidism with a frequency of 1.5–1.75 percent in secondary hyperparathyroidism and 3–4 percent in primary hyperparathyroidism.

It usually affects young people especially females with varying degrees of aggressiveness and risks of recurrence [1–3]. It can affect the base of the skull, orbits, paranasal sinuses, spinal column [4, 5] as well femur, tibia, humerus, clavicles and scapula. However, it is relatively rare in the maxilla with a frequency of 4.5–11.8 percent [6].

Radiologically, brown tumour in the jaws presents as a well-defined radiolucent osteolytic lesion that can encircle the roots of teeth, making it hard to differentiate it from other maxillary expansive lesions that can present with a similar imaging.

Histopathological examination of brown tumours can suggest the diagnosis but may not be sufficient to differentiate it conclusively from other lesions which can have similar microscopic and macroscopic features such as regenerative granuloma [4, 7, 8], giant cell granuloma, giant cell tumour, aneurysmatic cyst, cherubism, Paget's disease, odontologic bone tumour, and nonodontologic fibrous dysplasia. A final diagnosis can be defined only by evaluating the radiological findings with histopathological, laboratory, and clinical data. At present, brown tumour is considered as a reparative cellular process rather than a real neoplasia. This phenomenon is considered as pathognomonic of hyperparathyroidism secondary to renal failure, especially in patients on long-term hemodialysis [9–13].

Usually brown tumours do not present with pain unless their dimensions are large enough to compress neighbouring nerve structures. They are often discovered incidentally on routine radiological examinations. In most cases, when the pathway of growth of the tumour is closed and its position is not causing clinical problems, its surgical treatment is not indicated nor necessary.

The most important complications of this neoformation are related to its position and size and the possible effects on nearby structures. It can be a cause of an increased risk of fracture, of spinal cord compression, or of facial disfiguration, compromising normal functions such as mastication, compression, phonation, and social ease of the patient. Other neurological complications include diplopia if the optical nerve is involved [5, 14].

The clinical management of a brown tumour aims primarily to reduce the elevated parathyroid hormone levels by pharmacological treatment. Surgical treatment is reserved to nonresponders or to patients with painful symptomatology or alteration of normal function.

## 2. Case Report

**Figure 1:**
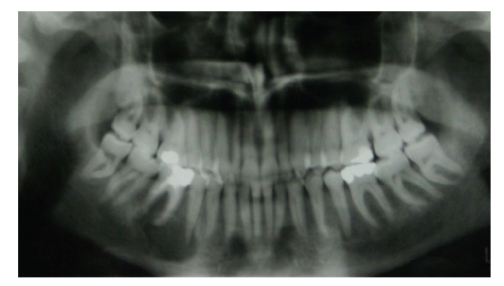
(December 12, 2000) X-ray of the maxilla that highlighted a bony rarefaction area.

In this paper, we describe a 40-years-old woman with a local lesion of the right side of the maxilla. Her history reveals that she was in relatively good health until the age of 20, when she started to complain of frequent headaches and vomiting up to three times a week. Clinical evaluation revealed arterial hypertension and renal failure. A kidney biopsy was not diagnostic. In February 1989, she was started on chronic haemodialysis three times weekly. In 1991, she received a kidney transplant from a cadaveric donor. In 1997, she developed a non-Hodgkin lymphoma. Chemotherapy for one year lead to a complete remission of the lymphoma. In March 1999, she developed aseptic necrosis of the femoral head which was treated surgically. In June 1999, as she developed progressive deterioration of the transplanted kidney function, haemodialysis three times weekly was restarted. In October 1999, the transplanted kidney was removed and during surgery, she developed thrombosis of the left central retinal vein. Laboratory investigations in December 2000 revealed elevated serum parathyroid hormone (PTH) level of 282 pg/mL (normal range 14–72 pg/mL). At the same time, she noted a swelling in the right side of the maxilla. A radiograph of the maxilla showed an area of bone rarefaction (Figure 1).

**Figure 2:**
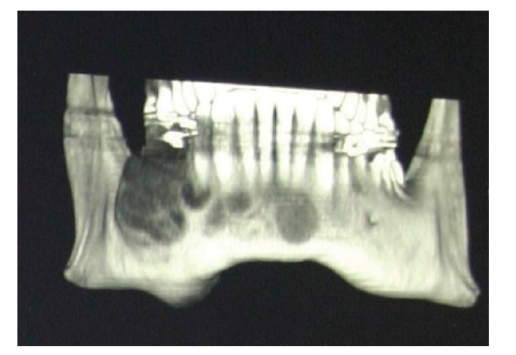
(May 29, 2001) TC deltascan shows the hypodense, disomogeneus borders neoformation with erosive character.

Five months later, a computed tomography and an electronic rebuilding dental scan showed a hypodense neo-formation with heterogeneous borders and erosions. Bone structure was interrupted both laterally and medially on the lingual side. A lesion with a maximum diameter of about 4 cm involved the roots and peri-apical tissue of teeth 4.4, 4.5, 4.6, and part of 4.7. Two other similar lesions each about 1 cm in diameter were seen in the chin region and the right side of the maxilla (Figure 2).

**Figure 3:**
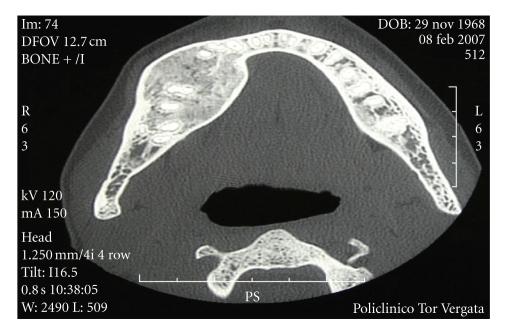
(February 08, 2007) TC deltascan reveals a thickening of alveolar bone of the right mandibular arch with a deformity of profile without a gap of cortical and reveals the regular morphology along the mandibular canal, without sure images referable to infiltration on it.

Since radiographs showed a radiographic image compatible with more than one possible lesion and hence were not sufficient to provide a definitive diagnosis, a biopsy of the lesions was performed. Histological examination revealed giant bone cells. This together with the laboratory evidence for secondary hyperparathyroidism, the diagnosis of a brown tumour was made. The patient was treated for secondary hyperparathyroidism with vitamin D, calcium, bisphosphonates, phosphate binders, and reduced phosphate intake. In July 2001, despite the above treatment, she continued to have severe and worsening hyperparathyroidism with PTH levels of 1700 pg/mL, with serum calcium of 10.5 mg/dl (normal range 8.6–10.2 mg/dl), phosphate of 5.3 mg/dl (normal range 2.7–4.5 mg/dl), and alkaline phosphatase of 319 IU/L (normal range 40–120 IU/L). At the same time, the bony lesions increased in size but with signs of modest recalcification. In October 2001, the refractoriness to medical therapy was evident and a parathyroidectomy became necessary. Surgical treatment consisted of removal of all 4 parathyroids and implantation of 1/3 of the inferior parathyroid in the right forearm muscle. This was followed by a dramatic fall in serum PTH levels and normalisation of serum calcium and phosphate levels resulting in regression of the brown tumour. February 2002, the lesions were partially calcified and in June 2003, computed tomography and dental scan showed a normal bone structure in the area that was previously involved with the brown tumour but with residual hyperostosis. In December 2003, alveolar bone was thickened both on the external and on the medial side. Thereafter, annual computed tomography and dental scan and monthly blood tests were carried out, and on the last examination in February 2007, there was thickened alveolar bone of the right mandibular arch with a profile deformity but without a cortical gap. There was a regular morphology of the mandibular canal without signs of infiltration (Figure 3).

In summary, this case shows that it is possible to avoid surgical of a large brown tumour even though it compromised the physical appearance of the patient and caused dysfunction of the masticatory apparatus. The surgical resolution of hyperparathyroidism was enough to correct the calcium-phosphate-PTH imbalance and to result in regression of the jaw lesions.

## 3. Conclusion

Brown tumour can develop very quickly as happened in this case. Medical treatment, even if appropriate can be insufficient to control the secondary hyperparathyroidism. Therefore, an early diagnosis and a timely parathyroidectomy in a medically resistant hyperparathyroidism are an optimal option to control the growth of the bony lesion and to avoid further weakening of bone structure and consequent increased risk of fractures, compression of contiguous structures, deformities, and functional alteration of other involved areas of the skeleton.
